# A model based on PT-INR and age serves as a promising predictor for evaluating mortality risk in patients with SARS-CoV-2 infection

**DOI:** 10.3389/fcimb.2025.1499154

**Published:** 2025-04-03

**Authors:** Yongjie Xu, Minjie Tang, Zhaopei Guo, Yanping Lin, Hongyan Guo, Fengling Fang, Lin Lin, Yue Shi, Lu Lai, Yan Pan, Xiangjun Tang, Weiquan You, Zishun Li, Jialin Song, Liang Wang, Weidong Cai, Ya Fu

**Affiliations:** ^1^ Department of Laboratory Medicine, The First Affiliated Hospital, Fujian Medical University, Fuzhou, China; ^2^ Department of Laboratory Medicine, National Regional Medical Center, Binhai Campus of the First Affiliated Hospital, Fujian Medical University, Fuzhou, China; ^3^ Fujian Key Laboratory of Laboratory Medicine, The First Affiliated Hospital, Fujian Medical University, Fuzhou, China; ^4^ Gene Diagnosis Research Center, The First Affiliated Hospital, Fujian Medical University, Fuzhou, China; ^5^ Department of Laboratory Medicine, National Reginal Medical Center, Binhai Campus of the First Affiliated Hospital, Fujian Medical University, Fuzhou, China; ^6^ Department of Blood Transfusion, The First Affiliated Hospital, Fujian Medical University, Fuzhou, China; ^7^ Department of Laboratory Medicine, The Third Hospital of Xiamen, Xiamen, China; ^8^ Medical Research Center, Fujian Maternity and Child Health Hospital, Fuzhou, Fujian, China; ^9^ Department of Hepatopancreatobiliary Surgery, The First Affiliated Hospital, Fujian Medical University, Fuzhou, China; ^10^ Department of Laboratory Medicine, the Affiliated Hospital of Putian University, Putian University, Putian, China

**Keywords:** PT-INR, age, predictor, mortality, SARS-CoV-2

## Abstract

COVID-19 caused by the coronavirus SARS-CoV-2 has resulted in a global pandemic. Considering some patients with COVID-19 rapidly develop respiratory distress and hypoxemia, early assessment of the prognosis for COVID-19 patients is important, yet there is currently a lack of research on a comprehensive multi-marker approach for disease prognosis assessment. Here, we utilized a large sample of hospitalized individuals with COVID-19 to systematically compare the clinical characteristics at admission and developed a nomogram model that was used to predict prognosis. In all cases, those with pneumonia, older age, and higher PT-INR had a poor prognosis. Besides, pneumonia patients with older age and higher PT-INR also had a poor prognosis. A nomogram model incorporating presence of pneumonia, age and PT-INR could evaluate the prognosis in all patients with SARS-CoV-2 infections well, while a nomogram model incorporating age and PT-INR could evaluate the prognosis in those with pneumonia well. Together, our study establishes a prognostic prediction model that aids in the timely identification of patients with poor prognosis and helps facilitate the improvement of treatment strategies in clinical practice in the future.

## Introduction

COVID-19, short for a novel coronavirus disease, is a severe acute respiratory syndrome caused by the coronavirus SARS-CoV-2, resulting in a global pandemic defined by the Director-General of the World Health Organization (WHO) on March 11, 2020 (Rothan and Byrareddy, 2020; Rubin et al., 2020). The pathogen of this disease belongs to the beta genus of coronaviruses and is mainly transmitted through respiratory droplets and close contact, and the general population is susceptible to it (Wang et al., 2020).

Although most patients have a good prognosis, some patients rapidly develop respiratory distress and hypoxemia after the onset of the disease, leading to the development of acute respiratory distress syndrome (ARDS), and even multiple organ failure, of which the mortality rate is relatively high (Gilbert et al., 2020). Based on the severity of the condition, COVID-19 can be classified into four categories: mild, moderate, severe, and critical. Early identification of potential severe cases, preventing the progression from mild or moderate to severe or critical, as well as assessing the prognosis and predicting the outcome of COVID-19, pose significant challenges in clinical practice.

Several indicators have been reported to be associated with the prognosis of COVID-19. Throughout the course of infection, the persistently low count of eosinophils could have fatal consequences (Chen et al., 2020; Zhao et al., 2021). Significantly elevated levels of IL-6 were associated with adverse clinical outcomes (Coomes and Haghbayan, 2020). Additionally, age, gender, and hypertension were also associated with the severity of the disease (Shi et al., 2020). Nevertheless, there is currently a lack of research on a comprehensive multi-marker approach for disease prognosis assessment. Although there are already various early warning scoring systems that can comprehensively assess the severity of patients’ conditions and predict clinical outcomes, such as the National Early Warning Score version 2 (NEWS2) and the Acute Physiology and Chronic Health Evaluation II (APACHE II) scoring systems, prognostic prediction models based on specific clinical indicators are equally important and effective. These models can serve as supplements to early warning scoring systems, thereby enhancing the accuracy of predictions.

Therefore, this study aims to utilize data from a large cohort of hospitalized COVID-19 patients to systematically compare the clinical characteristics of different prognostic groups at the time of admission. Our objective is to develop a standardized prognostic prediction model that incorporates patients’ age and PT-INR data upon admission, in order to provide accurate predictive capabilities for different prognostic groups. Through this research, we hope to establish an effective prognostic prediction tool that will assist clinicians in timely identifying patients with poor prognoses, ultimately improving treatment strategies in the future.

## Methods

### Study design and population

The study population included hospitalized patients diagnosed with COVID-19 at our hospital. The training cohort consisted of patients who were discharged or deceased between December 1, 2022, and January 31, 2023, while the validation cohort included patients who were discharged or deceased between April 1, 2023, and April 30, 2023. Data collection was conducted through the hospital’s electronic medical record system, and the relevant data were compiled into spreadsheets and reviewed by senior physicians. The collected information included the patients’ age, gender, diagnosis at the time of admission, presence of pneumonia, laboratory test results, and prognosis. For the sample size calculation, we set the significance level at 0.05, the statistical power at 0.80, the expected effect size at 0.8, and the loss percentage at 20%, resulting in a final sample size of 554 participants. According to the guidelines and standards set by the World Health Organization (WHO) regarding COVID-19 (O. World Health, 2021), the diagnostic criteria for confirmed cases include the presence of one of the following microbiological evidence based on suspected cases: 1) The patient exhibits acute respiratory infection symptoms, such as fever, cough, fatigue, shortness of breath, sore throat, muscle or joint pain, headache, and loss of smell or taste; 2) Positive results for the novel coronavirus nucleic acid via real-time fluorescent RT-PCR testing; 3) Viral gene sequencing showing high homology with known novel coronaviruses; 4) A history of contact with confirmed COVID-19 cases within the past 14 days, or travel or residence in areas experiencing severe outbreaks; 5) Chest X-ray or CT scans revealing pneumonia or other lung lesions associated with COVID-19. The criteria for excluding COVID-19 typically include the following aspects: 1) Clinical Symptoms: Patients who do not exhibit COVID-19-related symptoms (such as fever, cough, shortness of breath, fatigue, muscle or joint pain, sore throat, headache, loss of taste or smell, etc.) usually do not meet the criteria for a COVID-19 diagnosis. 2) Test Results: If PCR or rapid antigen test results are negative, and the patient’s symptoms or clinical history do not align with COVID-19, then a COVID-19 diagnosis should also be excluded. 3) Other Causes: If the patient’s symptoms can be explained by other known causes (such as influenza, other respiratory viral infections, or bacterial pneumonia), and relevant tests confirm this, then a diagnosis of COVID-19 can be ruled out. 4) Epidemiological Background: An absence of epidemiological risk factors for COVID-19 (such as no contact with confirmed cases or recent travel to areas with high incidence of the disease) can also serve as a basis for exclusion from the diagnosis. 5) Imaging Studies: If X-ray or CT imaging does not show typical lesions associated with COVID-19, the patient may also be considered for exclusion from a COVID-19 diagnosis. Based on the clinical treatment outcomes, we categorized the patients into four groups: Cured Group: Patients who had a complete resolution of their condition after clinical treatment. Improved Group: Patients who showed significant improvement in their symptoms following treatment. Unimproved Group: This group includes a portion of patients whose condition did not improve and actually worsened after treatment. In these cases, family members chose to discontinue treatment, leading to the patient’s discharge against medical advice. Deceased Group: Patients who passed away during the course of clinical treatment. In our study, patients were categorized into two groups based on their clinical outcomes following treatment: “Effective Treatment” and “Ineffective Treatment.” Patients classified as having received “Effective Treatment” were those who demonstrated significant improvement in symptoms and met the discharge criteria during our observation/data collection period (this includes patients from both the “Cured” and “Improved” groups). In contrast, “Ineffective Treatment” refers to patients who did not meet these criteria within the specified observation period (this includes patients from both the “Unimproved” and “Deceased” groups). The written consent was obtained from each patient and the study was approved by the Ethics Committee of the First Affiliated Hospital of Fujian Medical University. Ethics Committee Approval Number: MTCA, ECFAH of FMU〔2015〕No. 084-2.

### Nomogram model construction and prognosis evaluation

In the training cohort, the one-way analysis of variance (ANOVA) was used to compare the differences in various clinical indicators among the cured, improved, unimproved, and dead groups. Indicators with significantly statistical differences were further filtered by lasso regression and logistic regression analysis. We further simplified the complex logistic regression model into a visualized nomogram by using the rms package of R. Subsequently, the efficiency of the visualized nomogram was evaluated by calibration curve and receiver operating characteristic (ROC) curve. The calibration curve was used to compare the association between actual outcomes and predicted probabilities. The ROC curve was used to assess the discriminative ability of the nomogram and then the area under the curve (AUC). Nomogram scores are utilized to predict the patient’s 30-day clinical outcomes during hospitalization. Cumulative events of effective treatment within 30 days of hospitalization based on cox regression analysis were visualized by using the survminer and ggplot2 packages of R.

### Statistical analysis

Statistical differences were evaluated by one-way analysis of variance (ANOVA), chi-squared test (Fisher’s exact test was used when needed), lasso regression, logist regression, and cox regression with IBM SPSS Statistics software (Version 22.0.0; IBM, Armonk, New York, USA) and R (version 4.1.0 http://www.r-project.org). All *P* values were two-tailed. *P* < 0.05 was considered to be statistically significant.

## Results

### Clinical characteristics of COVID-19 patients

**Figure 1:**
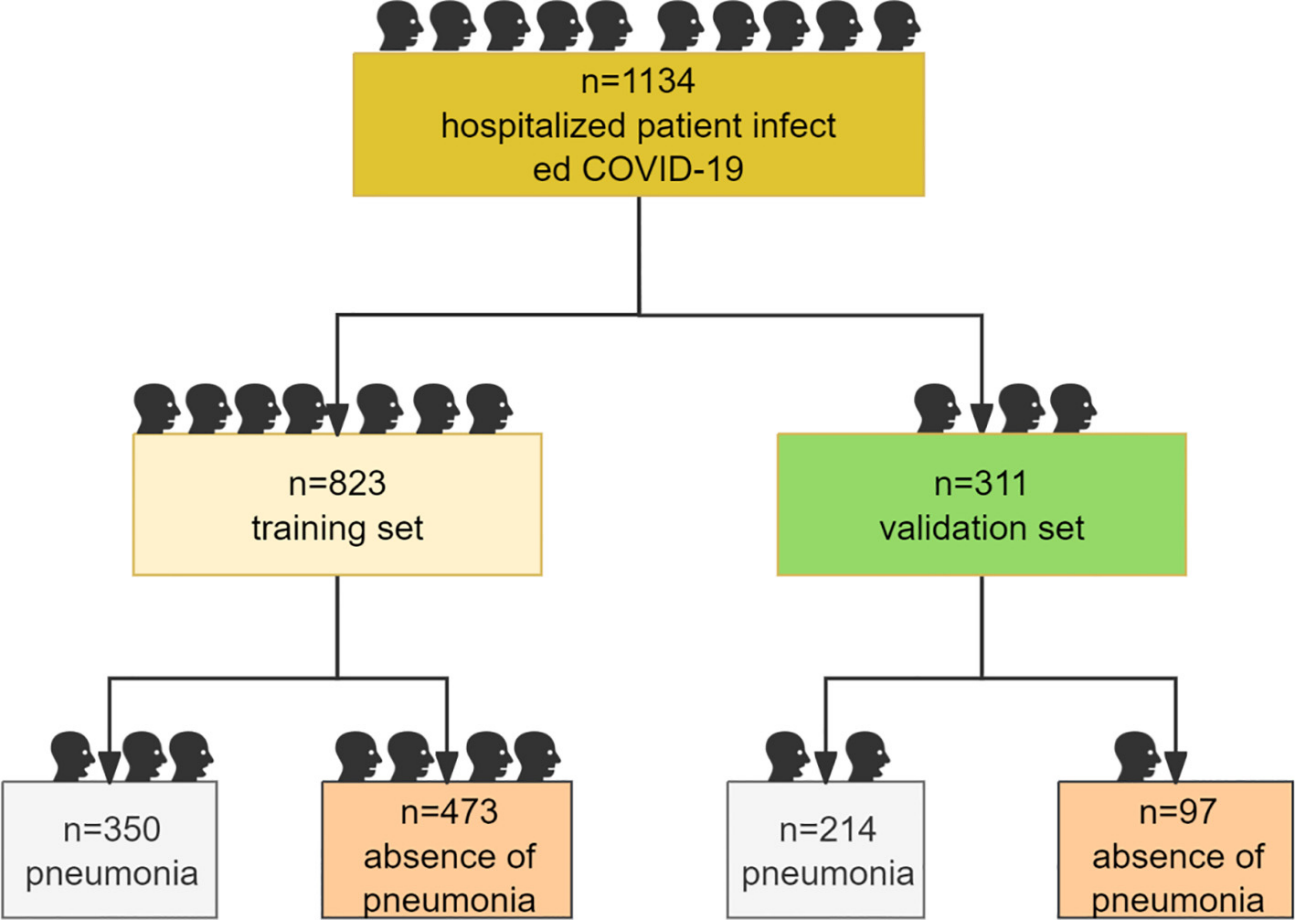
Study flow diagram.

The study enrolled a total of 1134 hospitalized patients who were infected with COVID-19, including 823 cases in the training set and 311 cases in the validation set (**Figure 1**). Among these 823 patients in the training set, 89 succumbed to the disease, 293 successfully recovered, while the remaining 441 individuals were still undergoing treatment at the time of their enrollment in the study. The clinical data of patients with different prognoses at admission in the training set are shown in **Table 1**. By anlysing the differences in the clinical characteristics among the cured, improved, unimproved and dead groups, significant differences in 38 indexes by ANOVA were observed across above 4 groups. The results shown that gender was associated with prognosis (*P* = 0.0297), and male patients have a poorer prognosis compared with female ones (Dead: male vs female, 73.03% vs 26.97%. Unimproved: male vs female, 70.59% vs 29.41%). Moreover, patients with pneumonia in this study also had the poorer prognosis compared with those without pneumonia (*P* < 0.0001. Dead: pneumonia vs non-pneumonia, 91.01% vs 8.99%). In addition, the elevated levels of 18 factors were found correlated to the poorer prognosis, while the declining levels of the other 18 factors were correlated to the poorer prognosis, In addition, the elevated levels of 18 biochemical and clinical factors were found to be correlated to the poorer prognosis as detailed in table.

**Table 1:** Clinical characteristics of patients infected with COVID-19.

Hospital discharge status	Cured (n = 293)	Improved (n = 424)	Unimproved (n = 17)	Dead (n = 89)	*χ^2^/F/U*	*P*
Gender					8.97	0.0297
male	167 (57.00%)	272 (65.15%)	12 (70.59%)	65 (73.03%)		
female	126 (43.00%)	152 (34.85%)	5 (29.41%)	24 (26.97%)		
Presence/Absence of pneumonia					140.62	<0.0001
yes	64 (21.84%)	199 (46.93%)	6 (35.29%)	81 (91.01%)		
no	229 (78.16%)	225 (53.07%)	11 (64.71%)	8 (8.99%)		
Age (year)	56.59 ± 17.68	62.97 ± 16.55	63.06 ± 16.28	73.42 ± 11.89	25.10	<0.0001
PT (s)	13.09 ± 5.98	13.18 ± 2.00	13.50 ± 2.70	15.04 ± 4.36	5.61	0.0008
PT-INR	1.09 ± 0.18	1.14 ± 0.20	1.17 ± 0.28	1.34 ± 0.48	25.52	<0.0001
APTT (s)	33.52 ± 7.86	34.87 ± 10.44	36.64 ± 12.25	41.47 ± 16.95	13.11	<0.0001
Fg (g/L)	3.64 ± 1.43	4.01 ± 1.55	3.65 ± 1.09	4.51 ± 2.14	7.75	<0.0001
TT(s)	17.90 ± 4.79	17.95 ± 2.86	19.25 ± 3.56	19.39 ± 9.69	2.81	0.0387
WBC (10^9^/L)	7.96 ± 5.30	7.50 ± 3.69	10.03 ± 12.44	8.96 ± 4.45	3.66	0.0122
%NEUT (%)	69.62 ± 14.65	69.07 ± 14.01	59.80 ± 16.02	79.07 ± 12.32	15.89	<0.0001
#NEUT (10^9^/L)	5.67 ± 3.73	5.45 ± 3.58	6.20 ± 7.60	7.38 ± 4.40	6.31	0.0003
%LYMPH (%)	19.78 ± 12.36	20.14 ± 11.14	27.49 ± 14.09	13.03 ± 9.86	12.52	<0.0001
#LYMPH (10^9^/L)	1.51 ± 3.70	1.33 ± 0.77	2.05 ± 1.73	0.94 ± 0.64	1.92	0.1255
%MONO (%)	5.85 ± 2.46	5.81 ± 2.42	6.30 ± 2.20	4.84 ± 1.83	4.88	0.0023
#MONO (10^9^/L)	0.43 ± 0.24	0.40 ± 0.20	0.55 ± 0.56	0.42 ± 0.24	2.72	0.0433
%EOS (%)	1.20 (0.00-20.70)	1.35 (0.00-19.40)	1.70 (0.00-5.00)	0.30 (0.00-4.90)	20.84	<0.001
#EOS (10^9^/L)	0.08 (0.00-2.89)	0.09 (0.00-1.46)	0.12 (0.01-0.33)	0.02 (0.00-0.28)	20.35	<0.001
%BASO (%)	0.57 ± 0.64	0.58 ± 0.62	0.81 ± 0.89	0.45 ± 0.73	1.89	0.1303
#BASO (10^9^/L)	0.05 ± 0.14	0.04 ± 0.04	0.17 ± 0.53	0.04 ± 0.05	7.10	0.0001
%LUC (%)	2.47 ± 1.92	2.55 ± 2.20	3.94 ± 6.53	1.93 ± 1.64	4.42	0.0043
#LUC (10^9^/L)	0.19 ± 0.33	0.16 ± 0.10	1.07 ± 3.81	0.14 ± 0.09	13.84	<0.0001
RBC (10^12^/L)	4.01 ± 0.91	3.95 ± 0.86	4.05 ± 0.90	3.74 ± 0.89	2.13	0.0946
HGB (g/l)	120.19 ± 26.21	118.02 ± 25.37	122.24 ± 27.93	111.74 ± 24.44	2.62	0.0496
HCT	0.36 ± 0.08	0.36 ± 0.07	0.38 ± 0.08	0.34 ± 0.07	3.12	0.0255
MCV (fL)	91.70 ± 8.05	91.71 ± 7.57	93.09 ± 7.04	91.31 ± 8.41	0.26	0.8580
MCH (Pg)	30.23 ± 3.06	30.05 ± 2.97	30.05 ± 2.95	30.14 ± 2.91	0.24	0.8695
MCHC (g/l)	329.42 ± 15.17	327.25 ± 14.91	325.24 ± 9.60	330.19 ± 16.22	1.92	0.1244
RDW (%)	14.59 ± 2.34	14.77 ± 2.30	14.35 ± 1.95	14.43 ± 1.81	0.85	0.4679
PLT (10^9^/L)	226.21 ± 114.64	241.15 ± 121.44	228.47 ± 83.27	201.46 ± 92.75	3.20	0.0229
MPV (fL)	9.01 ± 1.34	9.00 ± 1.29	9.38 ± 1.34	9.42 ± 1.37	3.01	0.0294
PDW (fL)	52.34 ± 9.45	51.77 ± 9.42	54.46 ± 8.13	53.63 ± 9.86	1.31	0.2698
PCT	0.20 ± 0.09	0.21 ± 0.10	0.21 ± 0.08	0.18 ± 0.08	2.49	0.0589
MPC (g/l)	255.29 ± 20.30	254.58 ± 19.69	251.53 ± 17.20	249.28 ± 20.23	2.25	0.0810
TBIL (μmol/L)	11.76 ± 17.99	12.92 ± 28.02	9.41 ± 5.99	10.17 ± 12.28	0.48	0.6960
DBIL (μmol/L)	6.33 ± 16.11	8.00 ± 25.17	4.69 ± 4.22	5.59 ± 10.41	0.63	0.5965
IBIL (μmol/L)	5.70 ± 5.67	4.93 ± 4.91	4.72 ± 3.38	4.58 ± 4.10	1.81	0.1445
TP (g/L)	64.71 ± 8.83	63.35 ± 8.92	62.45 ± 6.47	59.59 ± 8.53	7.79	<0.0001
ALB (g/L)	38.56 ± 6.58	37.59 ± 6.90	37.43 ± 6.27	34.03 ± 5.93	10.48	<0.0001
GLO (g/L)	26.09 ± 7.02	25.76 ± 4.99	25.02 ± 3.80	25.56 ± 5.26	0.39	0.7631
A/G	1.62 ± 1.21	1.51 ± 0.36	1.54 ± 0.40	1.38 ± 0.35	2.51	0.0575
ALT (U/L)	17.00 (1.00-227.00)	18.00 (1.00-1210.00)	16.00 (5.00-56.00)	19.00 (1.00-999.00)	2.21	0.137
AST (U/L)	21.00 (1.00-235.00)	21.00 (6.00-773.00)	19.00 (12.00-58.00)	26.00 (8.00-1714.00)	6.92	0.009
ALT/AST	1.05 ± 2.74	0.88 ± 0.47	0.80 ± 0.40	0.86 ± 0.59	0.66	0.5764
GGT (U/L)	54.62 ± 91.10	65.35 ± 121.95	26.82 ± 17.43	47.28 ± 54.88	1.60	0.1891
LDH (U/L)	194.00 (97.00-757.00)	193.50 (87.00-5425.00)	186.00 (126.00-1131.00)	243.00 (123.00-2246.00)	19.92	<0.001
ALP (U/L)	96.99 ± 103.30	104.74 ± 121.36	83.00 ± 62.73	80.51 ± 33.96	1.43	0.2315
CK (U/L)	71.00 (5.00-6830.00)	72.50 (6.00-13938.00)	76.00 (30.00-453.00)	139.00 (11.00-14514.00)	6.73	0.009
CKMB (U/L)	14.00 (3.00-94.00)	14.00 (4.00-442.00)	13.00 (8.00-27.00)	16.00 (5.00-369.00)	12.85	<0.001
UREA (mmol/L)	6.73 ± 7.01	7.04 ± 6.20	6.54 ± 3.34	10.44 ± 9.51	7.11	0.0001
CREA (μmol/L)	70.00 (0.06-1477.00)	72.00 (27.00-1613.00)	85.00 (48.00-157.00)	70.00 (14.00-1273.00)	0.03	0.858
UREA/CREA	0.82 ± 12.66	0.08 ± 0.03	0.07 ± 0.03	0.09 ± 0.04	0.60	0.6158
UA (μmol/L)	305.75 ± 120.19	310.25 ± 129.92	329.54 ± 121.34	313.11 ± 149.77	0.25	0.8627
GLU (mmol/L)	6.45 ± 3.03	6.77 ± 3.55	6.38 ± 3.05	8.54 ± 4.49	8.43	<0.0001
TC (mmol/L)	4.11 ± 1.23	4.12 ± 1.24	3.91 ± 1.29	3.67 ± 1.22	3.49	0.0153
TG (mmol/L)	1.32 ± 0.80	1.40 ± 0.78	1.20 ± 0.39	1.46 ± 1.63	0.84	0.4727
HDL-C (mmol/L)	1.12 ± 0.39	1.07 ± 0.39	1.09 ± 0.45	1.03 ± 0.39	1.70	0.1661
HDL-C/TC	0.29 ± 0.21	0.27 ± 0.09	0.28 ± 0.09	0.29 ± 0.10	1.96	0.1186
LDL-C (mmol/L)	2.56 ± 1.02	2.56 ± 1.06	2.48 ± 1.08	2.18 ± 1.09	3.56	0.0141
VLDL-C (mmol/L)	0.60 ± 0.37	0.63 ± 0.35	0.54 ± 0.18	0.66 ± 0.74	0.77	0.5113
APOA1 (g/L)	1.14 ± 0.36	1.11 ± 0.36	1.12 ± 0.37	0.98 ± 0.36	4.08	0.0069
APOB (g/L)	0.91 ± 0.30	0.93 ± 0.29	0.86 ± 0.30	0.86 ± 0.33	1.89	0.1306
APOA1/APOB	1.36 ± 0.56	1.29 ± 0.56	1.40 ± 0.56	1.26 ± 0.65	1.35	0.2559
CA (mmol/L)	2.19 ± 0.19	2.17 ± 0.20	2.18 ± 0.15	2.07 ± 0.16	9.65	<0.0001
P (mmol/L)	1.15 ± 0.31	1.11 ± 0.33	1.09 ± 0.27	1.15 ± 0.50	0.83	0.4778
MG (mmol/L)	1.01 ± 1.73	0.91 ± 0.11	0.95 ± 0.08	0.92 ± 0.15	0.56	0.6437
CO2CP (mmol/L)	24.81 ± 3.57	24.65 ± 4.25	24.84 ± 3.24	22.64 ± 4.40	7.17	<0.0001
K (mmol/L)	6.02 ± 9.35	5.60 ± 4.96	6.73 ± 6.44	5.14 ± 3.53	0.58	0.6283
NA (mmol/L)	130.96 ± 33.76	131.51 ± 33.53	124.25 ± 45.13	132.51 ± 28.71	0.31	0.8206
CL (mmol/L)	103.93 ± 11.48	104.90 ± 11.12	108.56 ± 13.61	103.80 ± 11.55	1.26	0.2856
AG (mmol/L)	19.24 ± 23.07	18.92 ± 22.66	22.82 ± 32.74	18.11 ± 19.46	0.22	0.8836
GFR (ml/min/1.73m^2^)	86.62 ± 30.01	78.97 ± 31.93	68.59 ± 34.24	73.84 ± 36.85	2.57	0.0529

numerical variables are represented using the mean ± standard deviation.PT, prothrombin time; PT-INR, prothrombin time-international normalized ratio; APTT, activated partial thromboplastin time; Fg, fibrinogen; TT, prothrombin time; WBC, white blood cell; %NEUT, percentage of neutrophils; #NEUT, neutrophil count; %LYMPH, percentage of lymphocytes; #LYMPH, lymphocyte count; %MONO, percentage of mononuclear cells; #MONO, monocyte count.; %EO, eosinophil percentage; #EO, eosinophil count; %BASO, basophil percentage; #BASO, basophil count; %LUC, percentage of unstained macrophages; #LUC, unstained macrophage count; RBC, red blood cell; HGB, hemoglobin; HCT, hematocrit; MCV, mean erythrocyte volume; MCH, mean erythrocyte hemoglobin volume; MCHC, mean erythrocyte hemoglobin concentration; RDW, erythrocyte distribution width; PLT, platelet; MPV, mean platelet volume; PDW, platelet distribution width; PCT, platelet specific volume; MPC, mean platelet component concentration; TBIL, total bilirubin; DBIL, direct bilirubin; IBIL, indirect bilirubin; TP, total protein; ALB, albumin; GLO, globulin; A/G, albumin-to-globulin ratio; ALT, alanine aminotransferase; AST, aspartate aminotransferase; ALT/AST, alanine aminotransferase to aspartate aminotransferase ratio; GGT, gamma-glutaminyl transferase; LDH, lactate dehydrogenase; ALP, alkaline phosphatase; CK, creatine kinase; CKMB, creatine kinase isoenzyme; UREA, urea; CREA, creatinine; UREA/CREA, urea to creatinine ratio; UA, uric acid; GLU, glucose; TC, total cholesterol; TG, triglyceride; HDL-C, high-density lipoprotein cholesterol; HDL-C/TC, ratio of high-density lipoprotein cholesterol to total cholesterol; LDL-C, low-density lipoprotein cholesterol; VLDL-C, very low-density lipoprotein cholesterol; APOA1, apolipoprotein A1; APOB, apolipoprotein B; APOA1/APOB, apolipoprotein A1 to apolipoprotein B ratio; CA, calcium; P, phosphorus; MG, magnesium; CO2CP, carbon dioxide binding capacity; K, potassium; NA, sodium; CL, chloride; AG, anion gap; GFR. Glomerular filtration rate.

### Presence/Absence of pneumonia, age and PT-INR at admission are potential predictors for prognosis post COVID-19 infection

**Figure 2:**
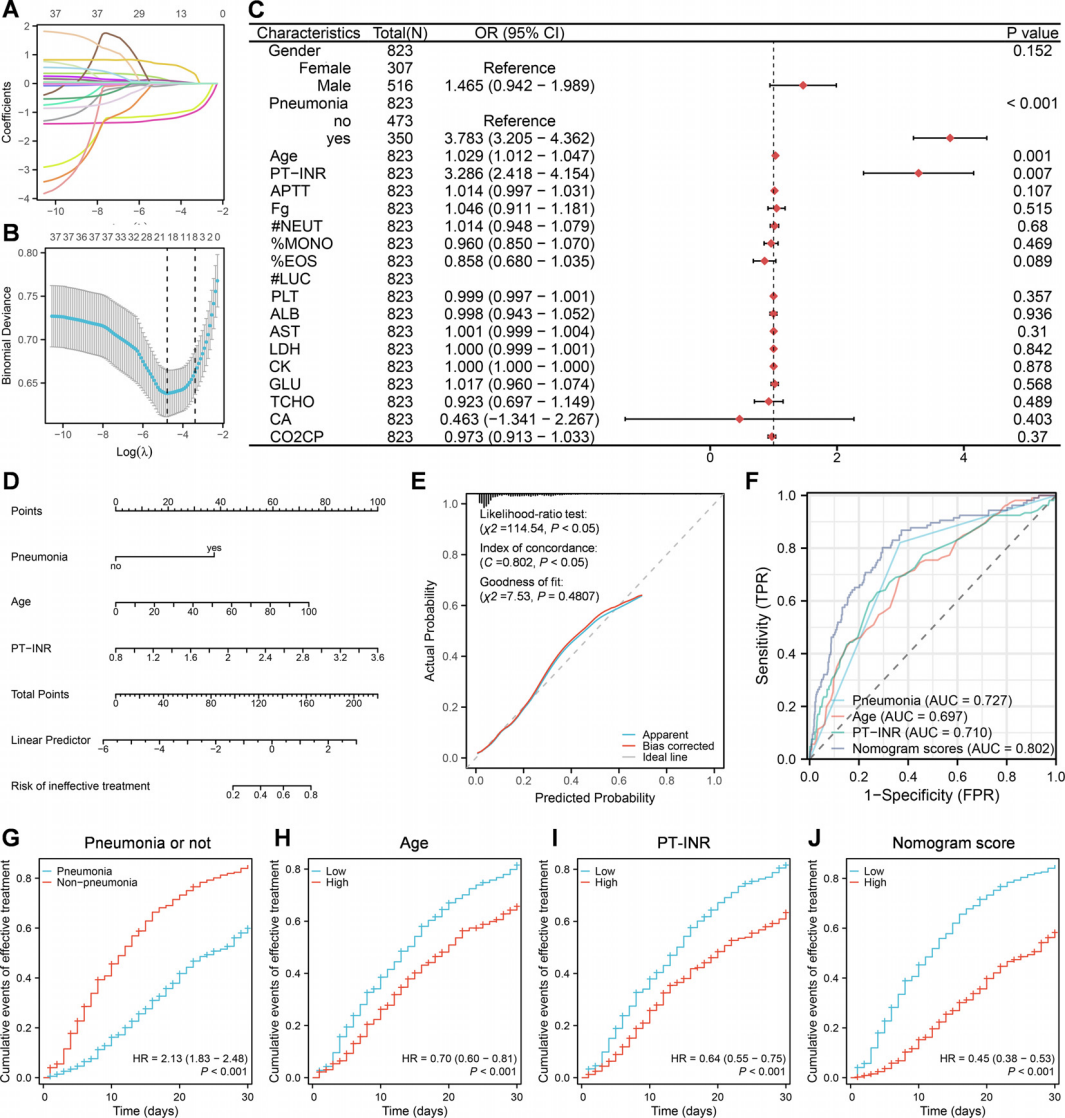
A nomogram model built based on pneumonia, age and PT-INR at admission for predicting prognosis post COVID-19 infection. **(A, B)** Lasso regression analysis. **(C)** Multivariate logistic regression analysis. **(D)** Construction of nomogram model based on multivariate logistic regression analysis. **(E)** Construction of calibration curves for the nomogram model. **(F)** Construction of ROC curves for nomogram scores, presence/absence of pneumonia, age, and PT-INR, respectively. **(G-J)** Cumulative events of effective treatment within 30 days of hospitalization based on cox regression analysis.

To evaluate factors associated with prognosis post COVID-19 infection, patients in this study were classified into effective-treatment group versus ineffective-treatment group. The effective group included cured and improved patients, while the ineffective group included unimproved and dead patients. One case was excluded from the analysis due to missing the data about duration of hospitalization. The lasso regression was used to initially screen the prognosis-related factors from above 38 indexes with significant difference among the cured, improved, unimproved and dead patients (**Table 1**, **Figures 2A, B**). Consequently, 19 of 38 indexes were selected for the subsequent analysis, including gender, presence/absence of pneumonia, age, PT-INR, APTT, Fg, #NEUT, %MONO, %EOS, #LUC, PLT, ALB, AST, LDH, CK, GLU, TCHO, CA, CO_2_CP. The logist regression was employed to analyze the probability of using these 19 indexes for predicting the prognosis. It was shown that except #LUC, 18 of 19 indexed were all significantly different between effective-treatment and ineffective-treatment groups by univariate analysis of cox regression, while only 3 indexes —— presence/absence of pneumonia(mean OR = 3.783, *P* < 0.001), age (mean OR = 1.029, *P* = 0.001) and PT-INR (mean OR = 3.286, *P* = 0.007) —— were significantly different between effective-treatment and ineffective-treatment groups by multivariate analysis of cox regression (**Figure 2C** **).** Further, these aforementioned 3 predictors were integrated to develop a nomogram model that could be used to evaluate the prognosis (**Figure 2D**). The observed and predicted values of this model exhibited a high level of agreement, indicating a reliable performance of the model (**Figure 2E**). Using the scores marked by the nomogram model, the receiver operating characteristic (ROC) curves were generated between the effective-treatment and ineffective-treatment groups (**Figure 2F**). The areas under the curve (AUC) of model scores, presence/absence of pneumonia, age and PT-INR were respectively 0.802, 0.727, 0.697 and 0.710, suggesting that the model could effectively predict the prognosis post COVID-19 infection. The cox regression analysis was employed to further enhance the reliability of the model in predicting patient prognosis by evaluating the cumulative events of treatment effectiveness within 30 days of hospitalization (**Figures 2G-J**). The findings indicated that younger age (low vs high, mean HR = 0.70, *P* < 0.001), lower PT-INR levels (low vs high, mean HR = 0.64, *P* < 0.001), and the absence of pneumonia (pneumonia vs absence of pneumonia, mean HR = 2.13 or absence of pneumonia vs pneumonia, mean HR = 1/2.13 = 0.47, *P* < 0.001) were correlated with a more favorable prognosis within 30 days of hospitalization. Furthermore, patients with lower model scores also exhibited improved outcomes within 30 days of hospitalization (low vs high, mean HR = 0.45, *P* < 0.001).

### Age and PT-INR at admission are potential predictors for prognosis in patients with pneumonia post COVID-19 infection

**Figure 3:**
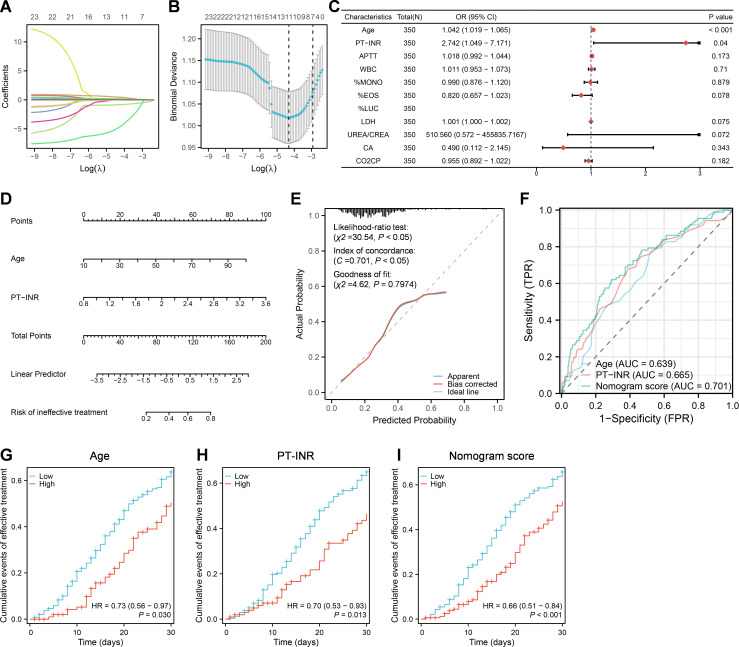
A nomogram model built based on age and PT-INR at admission for predicting prognosis post COVID-19 infection in patients with pneumonia. ***(*A, B*)*** Lasso regression analysis. **(C)** Multivariate logistic regression analysis. **(D)** Construction of nomogram model based on multivariate logistic regression analysis. **(E)** Construction of calibration curves for the nomogram model. **(F)** Construction of ROC curves for nomogram scores, age, and PT-INR, respectively. **(G-I)** Cumulative events of effective treatment within 30 days of hospitalization based on cox regression analysis.

To further obtain factors associated with recovery post COVID-19 infection in 350 patients with pneumonia, the differences in the clinical characteristics were analyzed among the cured, improved, unimproved and dead groups, and a total of 23 indicators were found to have statistically significant differences (**Table 2**). The lasso regression was also used to screen the recovery-related factors from aforementioned 23 indexes with significant difference by ANOVA among the cured group, uncured group and dead group (**Figures 3A, B**). 11 of 23 indexes were selected for the subsequent analysis, including age, PT-INR, APTT, WBC, %MONO, %EOS, %LUC, LDH, UREA/CREA, CA and CO_2_CP (**Figure 3C**). The logistic regression was used to analyze the feasibility of using these 11 indexes for assessing the probability of recovery post COVID-19 infection, and the results demonstrated that except #LUC, 10 of 11 indexed were all significantly different between effective-treatment and ineffective-treatment groups by univariate analysis of cox regression, while only 2 indexes; age (mean OR = 1.042, *P* < 0.001) and PT-INR (mean OR = 2.742, *P* = 0.04); were significantly different between effective-treatment and ineffective-treatment groups by multivariate analysis of cox regression (**Figure 3C** **).** Subsequently, a nomogram model incorporating age and PT-INR was constructed to assess the prognosis (**Figure 3D**). The calibration curve showed that the observed and predicted values of this model exhibited a high level of agreement, indicating a reliable performance of the model (**Figure 3E**). Using the scores marked by the nomogram model, ROC curves were generated between the effective-treatment and ineffective-treatment groups (**Figure 3F**). The areas under the curve (AUC) of model scores, age and PT-INR were respectively 0.701, 0.639 and 0.665. The cox regression analysis was also employed to evaluate the cumulative events of treatment effectiveness within 30 days of hospitalization in patients with pneumonia (**Figures 3G-I**). The findings indicated that younger age (low vs high, mean HR = 0.73, *P* = 0.030) and lower PT-INR levels (low vs high, mean HR = 0.70, *P* = 0.013) were correlated with a more favorable prognosis within 30 days of hospitalization in patients with pneumonia. Furthermore, pneumonia patients with lower model scores also exhibited improved outcomes within 30 days of hospitalization (low vs high, mean HR = 0.66, *P* < 0.001).

**Table 2:** Clinical characteristics of pneumonia patients infected with COVID-19.

Hospital discharge status	Cured (n = 64)	Improved (n = 199)	Unimproved (n = 6)	Dead (n = 81)	*χ^2^/F*	*P*
Gender					0.74	0.8640
male	43	139	5	57		
female	21	60	1	24		
Age (year)	62.31 ± 13.64	68.37 ± 15.05	75.33 ± 10.56	73.91 ± 11.40	8.71	<0.0001
PT (s)	13.42 ± 2.88	13.55 ± 2.30	16.03 ± 3.08	14.94 ± 4.52	5.46	0.0011
PT-INR	1.17 ± 0.31	1.18 ± 0.23	1.44 ± 0.33	1.33 ± 0.45	6.03	0.0005
APTT (s)	34.02 ± 8.30	36.08 ± 9.49	46.85 ± 15.88	40.39 ± 16.90	5.59	0.0009
Fg (g/L)	4.18 ± 1.76	4.33 ± 1.67	3.75 ± 1.16	4.67 ± 2.12	1.28	0.2813
TT(s)	18.90 ± 9.51	18.07 ± 2.81	21.70 ± 5.13	19.33 ± 10.14	1.19	0.3126
WBC (10^9^/L)	7.72 ± 3.68	7.86 ± 3.63	15.71 ± 19.31	9.00 ± 4.63	7.01	0.0001
%NEUT (%)	74.54 ± 12.59	72.50 ± 12.59	54.63 ± 15.30	78.91 ± 12.55	9.77	<0.0001
#NEUT (10^9^/L)	6.02 ± 3.64	5.93 ± 3.48	8.57 ± 10.38	7.41 ± 4.57	3.45	0.0168
%LYMPH (%)	15.94 ± 10.27	17.41 ± 9.73	29.73 ± 15.36	13.12 ± 10.12	7.13	0.0001
#LYMPH (10^9^/L)	1.02 ± 0.59	1.22 ± 0.70	3.32 ± 2.40	0.94 ± 0.66	21.11	<0.0001
%MONO (%)	5.49 ± 1.91	5.70 ± 2.66	6.83 ± 2.73	4.88 ± 1.85	3.00	0.0307
#MONO (10^9^/L)	0.41 ± 0.21	0.41 ± 0.22	0.86 ± 0.85	0.42 ± 0.25	6.76	0.0002
%EO (%)	1.39 ± 1.61	1.69 ± 2.19	1.42 ± 1.22	0.77 ± 1.04	4.70	0.0031
#EO (10^9^/L)	0.08 ± 0.09	0.12 ± 0.17	0.14 ± 0.11	0.05 ± 0.06	4.21	0.0061
%BASO (%)	0.39 ± 0.26	0.50 ± 0.36	1.23 ± 1.43	0.46 ± 0.75	5.47	0.0011
#BASO (10^9^/L)	0.03 ± 0.02	0.04 ± 0.04	0.41 ± 0.88	0.04 ± 0.05	21.68	<0.0001
%LUC (%)	2.24 ± 1.19	2.22 ± 1.17	6.80 ± 10.90	1.95 ± 1.72	12.76	<0.0001
#LUC (10^9^/L)	0.15 ± 0.07	0.15 ± 0.08	2.79 ± 6.40	0.14 ± 0.09	22.85	<0.0001
RBC (10^12^/L)	3.69 ± 1.00	3.90 ± 0.90	4.04 ± 1.06	3.77 ± 0.87	1.07	0.3616
HGB (g/l)	111.69 ± 28.52	116.70 ± 26.41	122.83 ± 39.21	112.90 ± 23.92	0.95	0.4155
HCT	0.34 ± 0.08	0.36 ± 0.08	0.38 ± 0.11	0.34 ± 0.07	1.47	0.2228
MCV (fL)	92.49 ± 8.01	91.78 ± 7.54	93.93 ± 10.12	91.36 ± 8.17	0.40	0.7560
MCH (Pg)	30.56 ± 3.06	30.13 ± 2.95	30.15 ± 3.52	30.19 ± 2.76	0.36	0.7806
MCHC (g/l)	330.20 ± 15.55	327.81 ± 15.53	320.83 ± 8.42	330.64 ± 16.05	1.34	0.2614
RDW (%)	14.60 ± 2.38	14.70 ± 2.16	15.25 ± 2.93	14.35 ± 1.71	0.69	0.5562
PLT (10^9^/L)	203.83 ± 90.68	228.82 ± 111.09	262.00 ± 84.23	205.73 ± 95.19	1.83	0.1406
MPV (fL)	8.95 ± 1.15	9.24 ± 1.41	10.32 ± 1.64	9.37 ± 1.35	2.47	0.0621
PDW (fL)	51.67 ± 10.81	53.00 ± 9.41	56.72 ± 8.38	53.74 ± 9.14	0.87	0.4596
PCT	0.18 ± 0.07	0.20 ± 0.09	0.27 ± 0.09	0.19 ± 0.08	3.24	0.0222
MPC (g/l)	254.45 ± 20.41	251.60 ± 19.42	244.67 ± 20.37	248.10 ± 20.00	1.49	0.2168
TBIL (μmol/L)	14.77 ± 22.92	11.12 ± 18.62	13.33 ± 7.34	9.59 ± 11.81	1.05	0.3714
DBIL (μmol/L)	9.04 ± 20.66	6.14 ± 14.94	7.63 ± 6.19	5.18 ± 10.08	0.85	0.4660
IBIL (μmol/L)	5.74 ± 4.01	4.98 ± 5.62	5.70 ± 3.85	4.42 ± 4.10	0.86	0.4599
TP (g/L)	63.10 ± 11.05	62.08 ± 8.88	62.92 ± 4.58	59.43 ± 8.07	2.33	0.0739
ALB (g/L)	36.75 ± 6.60	36.24 ± 6.90	37.88 ± 4.78	33.90 ± 5.75	3.19	0.0238
GLO (g/L)	26.35 ± 9.53	25.84 ± 4.77	25.03 ± 3.06	25.53 ± 5.22	0.26	0.8514
A/G	1.50 ± 0.40	1.44 ± 0.35	1.54 ± 0.30	1.38 ± 0.36	1.38	0.2485
ALT (U/L)	22.36 ± 18.13	30.69 ± 53.94	20.00 ± 18.80	49.78 ± 132.65	1.84	0.1390
AST (U/L)	27.84 ± 21.25	36.22 ± 45.90	26.50 ± 17.26	74.86 ± 250.85	2.27	0.0807
ALT/AST	0.86 ± 0.43	0.88 ± 0.50	0.83 ± 0.62	0.87 ± 0.60	0.05	0.9866
GGT (U/L)	57.20 ± 98.70	62.82 ± 112.37	38.33 ± 24.87	49.94 ± 56.75	0.42	0.7409
LDH (U/L)	232.67 ± 120.10	257.91 ± 159.38	335.17 ± 390.73	345.56 ± 342.67	4.28	0.0055
ALP (U/L)	93.53 ± 79.07	98.88 ± 95.72	114.17 ± 101.34	81.38 ± 35.13	0.98	0.4012
CK (U/L)	182.16 ± 405.56	357.27 ± 1227.93	130.83 ± 160.69	538.94 ± 1762.04	1.02	0.3838
CKMB (U/L)	18.67 ± 13.41	22.82 ± 39.19	15.50 ± 6.06	29.38 ± 51.20	1.05	0.3719
UREA (mmol/L)	7.86 ± 9.67	8.01 ± 7.15	6.10 ± 3.00	10.48 ± 9.24	2.14	0.0946
CREA (μmol/L)	105.97 ± 164.58	109.06 ± 123.96	98.00 ± 28.12	149.73 ± 213.14	1.50	0.2142
UREA/CREA	0.08 ± 0.04	0.08 ± 0.03	0.07 ± 0.03	0.09 ± 0.04	3.46	0.0166
UA (μmol/L)	309.25 ± 125.89	318.34 ± 143.07	345.07 ± 118.17	311.92 ± 150.50	0.17	0.9148
GLU (mmol/L)	7.03 ± 2.75	7.64 ± 4.49	6.12 ± 2.18	8.62 ± 4.58	2.11	0.0986
TC (mmol/L)	3.89 ± 1.30	4.04 ± 1.30	3.45 ± 1.18	3.71 ± 1.14	1.69	0.1690
TG (mmol/L)	1.24 ± 0.75	1.39 ± 0.69	1.22 ± 0.53	1.51 ± 1.69	0.85	0.4664
HDL-C (mmol/L)	1.00 ± 0.34	1.02 ± 0.39	0.91 ± 0.30	1.04 ± 0.38	0.29	0.8353
HDL-C/TC	0.27 ± 0.09	0.26 ± 0.09	0.27 ± 0.07	0.29 ± 0.10	1.51	0.2113
LDL-C (mmol/L)	2.49 ± 1.11	2.52 ± 1.10	2.18 ± 0.95	2.19 ± 1.04	1.95	0.1219
VLDL-C (mmol/L)	0.56 ± 0.34	0.63 ± 0.31	0.55 ± 0.24	0.68 ± 0.77	0.85	0.4680
APOA1 (g/L)	1.01 ± 0.35	1.05 ± 0.35	1.04 ± 0.31	0.99 ± 0.36	0.64	0.5931
APOB (g/L)	0.89 ± 0.32	0.93 ± 0.29	0.80 ± 0.29	0.87 ± 0.32	1.32	0.2687
APOA1/APOB	0.51 ± 0.06	0.52 ± 0.04	0.41 ± 0.17	0.65 ± 0.07	0.28	0.8372
CA (mmol/L)	2.13 ± 0.18	2.15 ± 0.22	2.20 ± 0.13	2.07 ± 0.15	3.39	0.0183
P (mmol/L)	1.12 ± 0.34	1.09 ± 0.36	1.10 ± 0.25	1.14 ± 0.48	0.37	0.7739
MG (mmol/L)	0.90 ± 0.13	0.92 ± 0.12	0.91 ± 0.04	0.92 ± 0.15	0.46	0.7095
CO2CP (mmol/L)	24.73 ± 3.60	24.14 ± 4.13	24.08 ± 3.66	22.62 ± 4.23	3.81	0.0105
K (mmol/L)	5.82 ± 5.53	5.60 ± 4.80	4.63 ± 0.64	5.23 ± 3.69	0.28	0.8398
NA (mmol/L)	129.46 ± 37.06	131.48 ± 33.99	139.22 ± 2.57	131.84 ± 29.99	0.19	0.9061
CL (mmol/L)	104.97 ± 10.94	105.33 ± 11.70	103.23 ± 3.93	103.89 ± 12.05	0.34	0.7959
AG (mmol/L)	19.97 ± 24.16	19.64 ± 23.67	11.90 ± 3.42	18.56 ± 20.27	0.27	0.8459
GFR (ml/min/1.73m^2^)	77.16 ± 32.59	75.00 ± 32.61	67.45 ± 17.94	73.03 ± 37.13	0.28	0.8403

numerical variables are represented using the mean ± standard deviation. PT, prothrombin time; PT-INR, prothrombin time-international normalized ratio; APTT, activated partial thromboplastin time; Fg, fibrinogen; TT, prothrombin time; WBC, white blood cell; %NEUT, percentage of neutrophils; #NEUT, neutrophil count; %LYMPH, percentage of lymphocytes; #LYMPH, lymphocyte count; %MONO, percentage of mononuclear cells; #MONO, monocyte count.; %EO, eosinophil percentage; #EO, eosinophil count; %BASO, basophil percentage; #BASO, basophil count; %LUC, percentage of unstained macrophages; #LUC, unstained macrophage count; RBC, red blood cell; HGB, hemoglobin; HCT, hematocrit; MCV, mean erythrocyte volume; MCH, mean erythrocyte hemoglobin volume; MCHC, mean erythrocyte hemoglobin concentration; RDW, erythrocyte distribution width; PLT, platelet; MPV, mean platelet volume; PDW, platelet distribution width; PCT, platelet specific volume; MPC, mean platelet component concentration; TBIL, total bilirubin; DBIL, direct bilirubin; IBIL, indirect bilirubin; TP, total protein; ALB, albumin; GLO, globulin; A/G, albumin-to-globulin ratio; ALT, alanine aminotransferase; AST, aspartate aminotransferase; ALT/AST, alanine aminotransferase to aspartate aminotransferase ratio; GGT, gamma-glutaminyl transferase; LDH, lactate dehydrogenase; ALP, alkaline phosphatase; CK, creatine kinase; CKMB, creatine kinase isoenzyme; UREA, urea; CREA, creatinine; UREA/CREA, urea to creatinine ratio; UA, uric acid; GLU, glucose; TC, total cholesterol; TG, triglyceride; HDL-C, high-density lipoprotein cholesterol; HDL-C/TC, ratio of high-density lipoprotein cholesterol to total cholesterol; LDL-C, low-density lipoprotein cholesterol; VLDL-C, very low-density lipoprotein cholesterol; APOA1, apolipoprotein A1; APOB, apolipoprotein B; APOA1/APOB, apolipoprotein A1 to apolipoprotein B ratio; CA, calcium; P, phosphorus; MG, magnesium; CO2CP, carbon dioxide binding capacity; K, potassium; NA, sodium; CL, chloride; AG, anion gap; GFR. Glomerular filtration rate.

### 30 The constructed nomogram model is validated for evaluating prognosis within 30 days of hospitalization in 311 patients post COVID-19 infection

**Figure 4:**
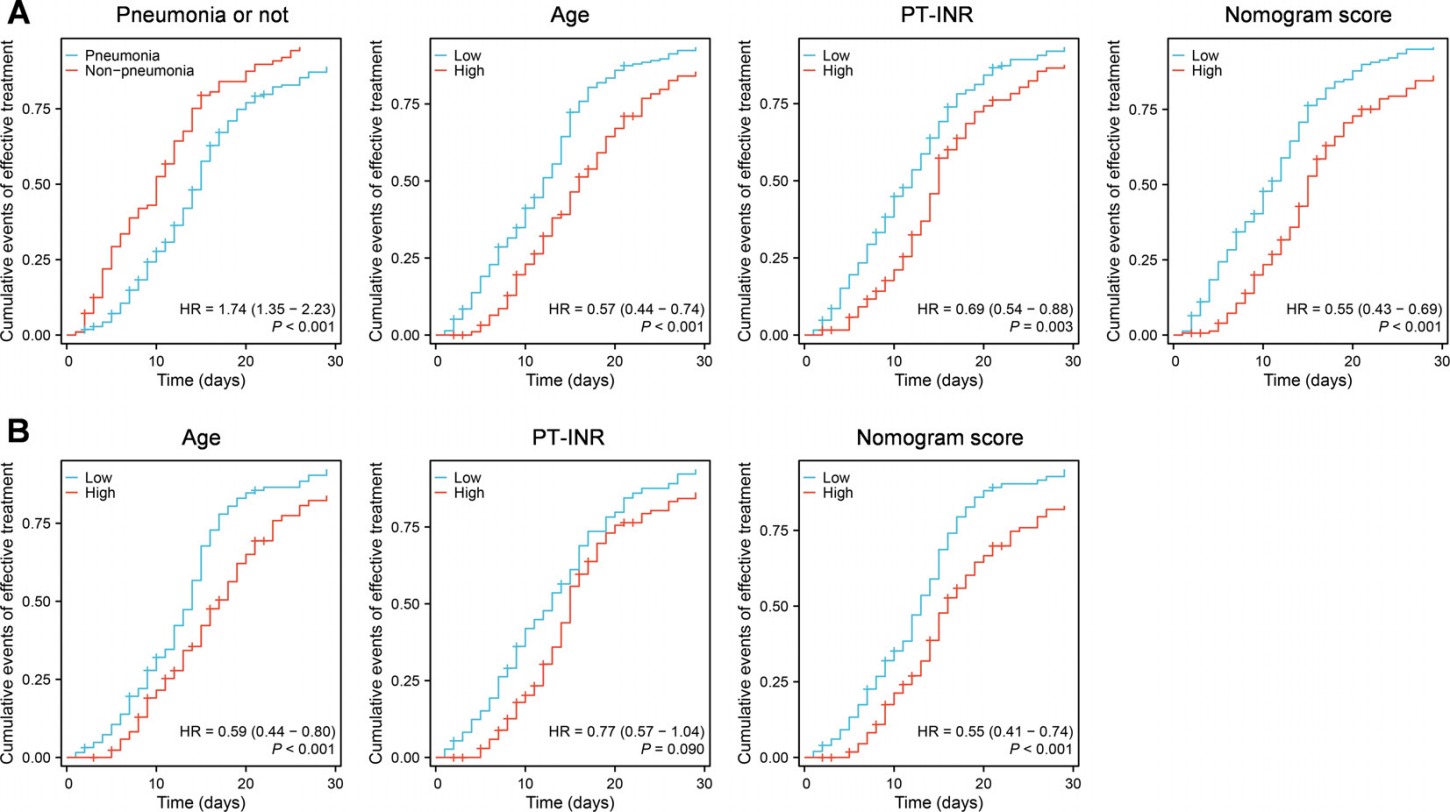
The validation of the constructed nomogram models for evaluating prognosis. **(A)** The validation of the constructed nomogram model incorporating the presence of presence/absence of pneumonia, age and PT-INR. **(B)** The validation of the constructed nomogram model incorporating age and PT-INR.

Furthermore, we utilized the constructed nomogram models to evaluate the prognosis of 311 patients in the validation set within 30 days of hospitalization. The cox regression analysis was employed to analyze prognosis by evaluating the cumulative events of treatment effectiveness within 30 days of hospitalization (**Figure 4**). The findings indicated that in all 311 patients, younger age (low vs high, mean HR = 0.57, *P* < 0.001), lower PT-INR levels (low vs high, mean HR = 0.69, *P* = 0.003), and the absence of pneumonia (pneumonia vs absence of pneumonia, mean HR = 1.74 or absence of pneumonia vs pneumonia, mean HR = 1/1.74 = 0.57, *P* < 0.001) were correlated with a more favorable prognosis within 30 days of hospitalization (**Figure 4A**). Furthermore, patients with lower model scores exhibited improved outcomes within 30 days of hospitalization (low vs high, mean HR = 0.54, *P* < 0.001) (**Figure 4A**). Moreover, in patients with pneumonia, except PT-INR, the younger age (low vs high, mean HR = 0.59, *P* < 0.001) and lower model scores (low vs high, mean HR = 0.55, *P* < 0.001) were also correlated with a more favorable prognosis within 30 days of hospitalization (**Figure 4B**).

## Discussion

The outbreak of coronavirus disease 2019 (COVID-19) caused by severe acute respiratory syndrome coronavirus 2 (SARS-CoV-2) has resulted in high morbidity and mortality rates worldwide. As of December 19, 2021, COVID-19 has impacted 273 million people and resulted in over 5.3 million deaths (Zhang et al., 2022). SARS-CoV-2 infection may initially be asymptomatic, until severe pneumonia, respiratory distress, organ dysfunction, and even death occur (Li et al., 2020), raising questions regarding the risks and protective factors of COVID-19. In this study, we utilized large-scale clinical data to compare the differences in clinical characteristics among different prognostic groups at admission and attempted to develop a model for predicting patient outcomes.

This study compared the clinical characteristics of different prognostic groups at admission and found a correlation between the presence of presence/absence of pneumonia, gender, age, and 35 laboratory biomarkers with clinical outcomes (**Table 1**). In addition, to further explore death - related factors, we divided the subjects in Tables 1 and 2 into a death group and a non-death group (including the cured, improved, and unimproved groups), and created Tables 3 and 4 as **Supplementary Materials**. Our current study demonstrated that older age, male gender, and the presence of pneumonia were closely associated with poor prognosis, consistent with previous research findings (Fauci et al., 2020). COVID-19 has been reported to cause coagulation dysfunction, characterized by significant elevation of D-dimer and fibrinogen, mild thrombocytopenia, and mild prolongation of PT/APTT (Lim and McRae, 2021). In this study, we found elevation of fibrinogen and PT-INR, reduction of PLT, and prolongation of PT, APTT and TT in patients with a poor prognosis. Some researchers have reported that COVID-19 could also cause alterations in hemogram of patients (Wang et al., 2020; Zhao et al., 2021). The current study displayed that significant reduction of %EOS, #EOS, %LYMPH, and %MONO but significant elevation of %NEUT and #NEUT at admission in dead cases. Besides, we also identified several biochemical indicators at admission, including TP, ALB, ALT, AST, LDH, CK, CKMB, UREA, CREA, GLU, TC, LDL-C, APOA1, CA, and CO_2_CP, that exhibited significant alterations in patients with adverse prognosis, particularly in fatal cases. These biochemical changes suggested that multi-organ involvement might be a major contributing factor to poor prognosis in patients. Together, these observations provide a detailed analysis of the clinical characteristic differences at admission among different prognostic groups, offering a data-driven support for clinicians in assessing the severity of patients’ conditions.

Based on the analysis of variance, lasso regression, and logistic regression, we found that the presence of pneumonia, older age, and higher PT-INR at admission were the most important prognostic indicators for COVID-19 patients. Moreover, older age and higher PT-INR were also important prognostic indicators for COVID-19 patients with pneumonia. Although the prognostic indicators for patients with COVID-19 and pneumonia show limited predictive capability, with individual AUC values for age and PT-INR falling below 0.7, our study demonstrates that by constructing a multivariable model that incorporates both age and PT-INR, the overall predictive ability of the model exceeds 0.7. This indicates that while the predictive power of certain individual factors may be limited, their combination in a multivariable model can significantly enhance predictive performance. This underscores the importance of utilizing multivariable models in clinical predictions. Clearly, existing studies have shown that the presence of pneumonia is one of the main causes of mortality in COVID-19 patients (Wiersinga et al., 2020; Zhang et al., 2022). The findings of O’Driscoll et al. demonstrated that in the population studied, the fatality rate of SARS-CoV-2 infection increased with age, beginning as early as 5 years old (O’Driscoll et al., 2021), which highlighted the significant influence of age on the prognosis of SARS-CoV-2 infection and was consistent with the experimental outcomes in our study. Similar to our findings, several studies reported a significant increase in PT-INR in COVID-19 patients with poor prognosis (Aminasnafi et al., 2022; Ceci et al., 2023; Wu et al., 2023), suggesting that liver involvement might be a major factor contributing to COVID-19 mortality. Collectively, these findings highlight the close relationship between age, PT-INR, and poor prognosis in COVID-19 patients, and it is the first to establish a predictive model based on age and PT-INR. Several studies (Martin-Rodriguez et al., 2022; Wibisono et al., 2022) have demonstrated that the National Early Warning Score version 2 (NEWS2) can effectively predict clinical deterioration and hospitalization outcomes in COVID-19 patients, particularly in emergency and inpatient settings. Its simplicity and ease of use make it an ideal tool for initial screening in clinical practice. However, based on the analyses presented in **Supplementary Figures S1 and S2**, we found that the ROC score of the NEWS2 assessment from our observed case records was lower than that of our model, indicating that our study’s model has relatively good predictive performance. Our study has several limitations. It is currently a single-center retrospective study, and further validation of our model’s efficacy will require a multicenter approach. Additionally, due to incomplete data collection, we were unable to conduct a correlation analysis between the symptoms of COVID-19 patients and the severity of their condition.

In conclusion, this study emphasizes the strong correlation between age, PT-INR at hospital admission, and patient prognosis. The prognostic model developed based on age and PT-INR can effectively identify patients at higher risk of poor outcomes. Our findings enhance the understanding of COVID-19 disease characteristics and provide valuable guidance for clinical management and treatment.

## Data Availability

The raw data supporting the conclusions of this article will be made available by the authors, without undue reservation.
