# Supplementary material for: A model based on PT-INR and age serves as a promising predictor for evaluating mortality risk in patients with SARS-CoV-2 infection
Source: Front Cell Infect Microbiol. 2025 Apr 3;15:1499154. doi: 10.3389/fcimb.2025.1499154 (PMC12003402; doi:10.3389/fcimb.2025.1499154)
Supplement: Supplementary file 1 [file Table1.docx]

**Table 3. Clinical charateristics of patients infected with COVID-19**

| **Hospital discharge status** | **total patients（n=823）** | **Undead（n=734）** | **Dead (n = 89)** | **t/*χ^2^*/U** | **P** |
| --- | --- | --- | --- | --- | --- |
| Gender |  |  |  | 4.559 | 0.033 |
| male | 516（62.70%） | 451（54.80%） | 65（7.90%） |  |  |
| female | 307（37.30%） | 283（34.38%） | 24（2.92%） |  |  |
| Presence/Absence of pneumonia |  |  |  | 95.975 | <0.001 |
| yes | 350(42.53%) | 269(32.69%) | 81(9.84%) |  |  |
| no | 473(57.47%) | 465(56.50%) | 8(0.97%) |  |  |
| Age (year) | 61.83±0.06 | 60.43±17.26 | 73.42±11.89 | -9.199 | 0.000 |
| PT | 13.36±4.16 | 13.15±4.09 | 15.04±4.36 | -3.883 | 0.000 |
| PTINR | 1.14±0.24 | 1.12±0.20 | 1.34±0.44 | -4.638 | 0.000 |
| APTT (s) | 35.14±10.82 | 34.37±9.56 | 41.47±16.95 | -3.88 | 0.000 |
| Fg (g/L) | 3.93±1.60 | 3.86±1.50 | 4.51±2.14 | -2.783 | 0.006 |
| TT（s） | 18.12±4.78 | 17.96±3.76 | 19.39±9.69 | -1.378 | 0.172 |
| WBC (10^9^/L) | 7.87±4.74 | 7.74±4.76 | 8.96±4.45 | -2.296 | 0.022 |
| %NEUT (%) | 70.16±14.48 | 69.07±14.37 | 79.07±12.23 | -6.292 | 0.000 |
| #NEUT (10^9^/L) | 5.75±3.88 | 5.55±3.77 | 7.38±4.40 | -4.231 | 0.000 |
| %LYMPH (%) | 19.39±11.77 | 20.16±11.75 | 13.03±9.86 | 5.493 | 0.000 |
| %MONO (%) | 5.73±2.39 | 5.83±2.43 | 4.84±1.83 | 3.736 | 0.000 |
| %EOS (%) | 1.10（0.00，20.70） | 1.84±2.13 | 0.76±1.02 | 19.83 | <0.001 |
| #EOS (10^9^/L) | 0.08（0.00，2.89） | 0.12±0.19 | 0.05±0.06 | 19.35 | <0.001 |
| %LUC (%) | 2.49±2.23 | 2.55±2.29 | 1.93±1.64 | 2.502 | 0.013 |
| RBC (10^12^/L) | 3.95±0.88 | 3.98±0.88 | 3.74±0.89 | 2.348 | 0.019 |
| HGB (g/l) | 118.20±25.70 | 118.99±25.76 | 111.74±24.44 | 2.519 | 0.012 |
| HCT | 0.36±0.07 | 0.36±0.07 | 0.34±0.07 | 2.843 | 0.005 |
| PLT (10^9^/L) | 231.28±116.06 | 234.89±118.11 | 201.46±92.75 | 2.575 | 0.010 |
| MPV (fL) | 9.06±1.32 | 9.01±1.31 | 9.42±1.37 | -2.765 | 0.006 |
| PCT | 0.20±0.09 | 0.20±0.10 | 0.18±0.08 | 2.032 | 0.042 |
| MPC (g/l) | 9.06±1.32 | 254.79±19.87 | 249.28±20.23 | 2.465 | 0.014 |
| TP (g/L) | 63.41±8.91 | 63.87±8.85 | 59.59±8.53 | 4.328 | 0.000 |
| ALB (g/L) | 37.55±6.79 | 37.97±6.77 | 34.03±5.93 | 5.253 | 0.000 |
| A/G | 1.53±0.78 | 1.55±0.82 | 1.38±0.35 | 1.941 | 0.053 |
| ALT (U/L) | 18.00（1.00，1210.00） | 27.47±56.10 | 46.83±126.86 | 2.21 | 0.137 |
| AST (U/L) | 22.00（1.00，1714.00） | 32.25±46.69 | 70.57±239.64 | 6.34 | 0.012 |
| LDH (U/L) | 198.00（87.00，5425.00） | 240.29±231.95 | 337.04±328.37 | 18.93 | <0.001 |
| CK (U/L) | 73.00（5.00，14514.00） | 209.09±754.51 | 540.36±1704.30 | 6.16 | 0.013 |
| CKMB (U/L) | 14.00（3.00，442.00） | 19.47±29.82 | 28.53±49.09 | 12.06 | <0.001 |
| UREA (mmol/L) | 7.29±6.95 | 6.90±6.48 | 10.44±9.51 | -3.409 | 0.001 |
| CREA (μmol/L) | 71.00（0.06，1613.00） | 98.69±133.36 | 145.67±205.49 | 0.032 | 0.858 |
| GLU (mmol/L) | 6.84±3.53 | 6.63±3.34 | 8.54±4.49 | -3.874 | 0.000 |
| TC (mmol/L) | 4.06±1.24 | 4.11±1.23 | 3.67±1.22 | 3.166 | 0.002 |
| LDL-C (mmol/L) | 2.52±1.06 | 2.56±1.05 | 2.18±1.09 | 3.252 | 0.001 |
| APOA1 (g/L) | 1.10±0.36 | 1.12±0.36 | 0.98±0.36 | 3.353 | 0.001 |
| CA (mmol/L) | 2.17±0.20 | 2.18±0.20 | 2.07±0.16 | 5.264 | 0.000 |
| CO2CP (mmol/L) | 24.49±4.06 | 24.72±3.96 | 22.64±4.40 | 4.61 | 0.000 |

**Note.** numerical variables are represented using the mean ± standard deviation.PT, prothrombin time; PT-INR, prothrombin time-international normalized ratio; APTT, activated partial thromboplastin time; Fg, fibrinogen; TT, prothrombin time; WBC, white blood cell; %NEUT, percentage of neutrophils; #NEUT, neutrophil count; %LYMPH, percentage of lymphocytes; %MONO, percentage of mononuclear cells; %EO, eosinophil percentage; #EO, eosinophil count; %LUC, percentage of unstained macrophages; RBC, red blood cell; HGB, hemoglobin; HCT, hematocrit; PLT, platelet; MPV, mean platelet volume; PCT, platelet specific volume; MPC, mean platelet component concentration; TP, total protein; ALB, albumin; A/G, albumin-to-globulin ratio; ALT, alanine aminotransferase; AST, aspartate aminotransferase; LDH, lactate dehydrogenase; CK, creatine kinase; CKMB, creatine kinase isoenzyme; UREA, urea; CREA, creatinine; GLU, glucose; TC, total cholesterol; LDL-C, low-density lipoprotein cholesterol; APOA1, apolipoprotein A1; CA, calcium; CO2CP, carbon dioxide binding capacity.
