# Supplementary material for: A model based on PT-INR and age serves as a promising predictor for evaluating mortality risk in patients with SARS-CoV-2 infection
Source: Front Cell Infect Microbiol. 2025 Apr 3;15:1499154. doi: 10.3389/fcimb.2025.1499154 (PMC12003402; doi:10.3389/fcimb.2025.1499154)
Supplement: Supplementary file 2 [file Table2.docx]

**Table 4. Clinical charateristics of pneumonia patients infected with COVID-19**

| **Hospital discharge status** | **Total patients（n=350）** | **Undead（n=269）** | **Dead (n = 81)** | **t/*χ^2^*/U** | **P** |
| --- | --- | --- | --- | --- | --- |
| Gender |  |  |  | 0.146 | 0.884 |
| male | 244(69.71%) | 187(53.43%) | 57(16.29%) |  |  |
| female | 106(30.29%) | 82(23.43%) | 24(6.85%) |  |  |
| Age (year) | 68.66±14.42 | 67.08±14.87 | 73.91±11.40 | -4.386 | 0.001 |
| PT | 13.89±3.12 | 13.58±2.48 | 14.94±4.52 | -2.603 | 0.011 |
| PTINR | 1.22±0.32 | 1.18±0.26 | 1.33±0.45 | -2.781 | 0.007 |
| APTT (s) | 36.89±11.79 | 35.83±9.54 | 40.39±16.9 | -2.316 | 0.023 |
| Fg (g/L) | 4.37±1.79 | 4.28±1.68 | 4.67±2.12 | -1.721 | 0.086 |
| TT（s） | 18.57±6.73 | 18.35±5.29 | 19.33±10.14 | -1.148 | 0.252 |
| WBC (10^9^/L) | 8.23±4.63 | 8±4.61 | 9±4.63 | -1.706 | 0.089 |
| %NEUT (%) | 74.05±13.09 | 72.59±12.91 | 78.91±12.55 | -3.888 | 0.000 |
| #NEUT (10^9^/L) | 6.33±4 | 6.01±3.77 | 7.41±4.57 | -2.511 | 0.013 |
| %LYMPH (%) | 16.36±10.28 | 17.33±10.15 | 13.12±10.12 | 3.273 | 0.001 |
| %MONO (%) | 5.49±2.39 | 5.68±2.5 | 4.88±1.85 | 2.676 | 0.008 |
| %EOS (%) | 0.70(0.00,19.40) | 0.90(0.00,19.40) | 0.30(0.00,4.90) | 4.964 | 0.000 |
| #EOS (10^9^/L) | 0.05(0.00,1.46) | 0.06(0.00,1.46) | 0.02(0.00,0.28) | 4.672 | 0.000 |
| %LUC (%) | 2.24±1.95 | 2.33±2.01 | 1.95±1.72 | 1.544 | 0.124 |
| RBC (10^12^/L) | 3.83±0.92 | 3.85±0.93 | 3.77±0.87 | 0.681 | 0.496 |
| HGB (g/l) | 115.01±26.49 | 115.65±27.22 | 112.9±23.92 | 0.818 | 0.414 |
| HCT | 0.35±0.08 | 0.35±0.08 | 0.34±0.07 | 1.033 | 0.302 |
| PLT (10^9^/L) | 219.47±104.09 | 223.61±106.44 | 205.73±95.19 | 1.357 | 0.176 |
| MPV (fL) | 9.24±1.36 | 9.2±1.37 | 9.37±1.35 | -1.020 | 0.309 |
| PCT | 0.2±0.09 | 0.2±0.09 | 0.19±0.08 | 1.146 | 0.253 |
| MPC (g/l) | 251.19±19.79 | 252.12±19.67 | 248.1±19.99 | 1.608 | 0.109 |
| TP (g/L) | 61.67±9.15 | 62.34±9.36 | 59.43±8.07 | 2.527 | 0.012 |
| ALB (g/L) | 35.82±6.63 | 36.4±6.78 | 33.9±5.75 | 3.001 | 0.003 |
| A/G | 1.44±0.36 | 1.46±0.36 | 1.38±0.36 | 1.676 | 0.095 |
| ALT (U/L) | 19.00(1.00,999.00) | 18.00(1.00,678.00) | 20.00(1.00,999.00) | -1.419 | 0.160 |
| AST (U/L) | 23.00(3.00,1714.00) | 22.00(3.00,463.00) | 27.00(8.00,1714.00) | -1.460 | 0.148 |
| LDH (U/L) | 216.00(92.00,2246.00) | 208.00(92.00,1228.00) | 242.00(123.00,2246.00) | -2.340 | 0.021 |
| CK (U/L) | 84.50(5.00,14514.00) | 80.00(5.00,13938.00) | 139.00(11.00,14514.00) | -1.422 | 0.156 |
| CKMB (U/L) | 16.00(3.00,418.00) | 15.00(3.00,418.00) | 17.00(5.00,369.00) | -1.272 | 0.206 |
| UREA (mmol/L) | 8.52±8.17 | 7.93±7.75 | 10.48±9.24 | -2.253 | 0.026 |
| CREA (μmol/L) | 73.00(14.00,1299.00) | 74.00(27.00,1299.00) | 70.00(14.00,1273.00) | -1.664 | 0.099 |
| GLU (mmol/L) | 7.73±4.24 | 7.46±4.11 | 8.62±4.58 | -2.170 | 0.031 |
| TC (mmol/L) | 3.93±1.26 | 3.99±1.29 | 3.71±1.14 | 1.779 | 0.076 |
| LDL-C (mmol/L) | 2.43±1.09 | 2.5±1.1 | 2.19±1.04 | 2.299 | 0.022 |
| APOA1 (g/L) | 1.03±0.35 | 1.04±0.35 | 0.99±0.36 | 1.078 | 0.282 |
| CA (mmol/L) | 2.13±0.2 | 2.15±0.21 | 2.07±0.15 | 3.658 | 0.000 |
| CO2CP (mmol/L) | 23.9±4.11 | 24.28±4 | 22.62±4.23 | 3.225 | 0.001 |

**Note.** numerical variables are represented using the mean ± standard deviation.PT, prothrombin time; PT-INR, prothrombin time-international normalized ratio; APTT, activated partial thromboplastin time; Fg, fibrinogen; TT, prothrombin time; WBC, white blood cell; %NEUT, percentage of neutrophils; #NEUT, neutrophil count; %LYMPH, percentage of lymphocytes; %MONO, percentage of mononuclear cells; %EO, eosinophil percentage; #EO, eosinophil count; %LUC, percentage of unstained macrophages; RBC, red blood cell; HGB, hemoglobin; HCT, hematocrit; PLT, platelet; MPV, mean platelet volume; PCT, platelet specific volume; MPC, mean platelet component concentration; TP, total protein; ALB, albumin; A/G, albumin-to-globulin ratio; ALT, alanine aminotransferase; AST, aspartate aminotransferase; LDH, lactate dehydrogenase; CK, creatine kinase; CKMB, creatine kinase isoenzyme; UREA, urea; CREA, creatinine; GLU, glucose; TC, total cholesterol; LDL-C, low-density lipoprotein cholesterol; APOA1, apolipoprotein A1; CA, calcium; CO2CP, carbon dioxide binding capacity.
