# Supplementary material for: A model based on PT-INR and age serves as a promising predictor for evaluating mortality risk in patients with SARS-CoV-2 infection
Source: Front Cell Infect Microbiol. 2025 Apr 3;15:1499154. doi: 10.3389/fcimb.2025.1499154 (PMC12003402; doi:10.3389/fcimb.2025.1499154)
Supplement: Supplementary file 4 [file SupplementaryFile2.docx]

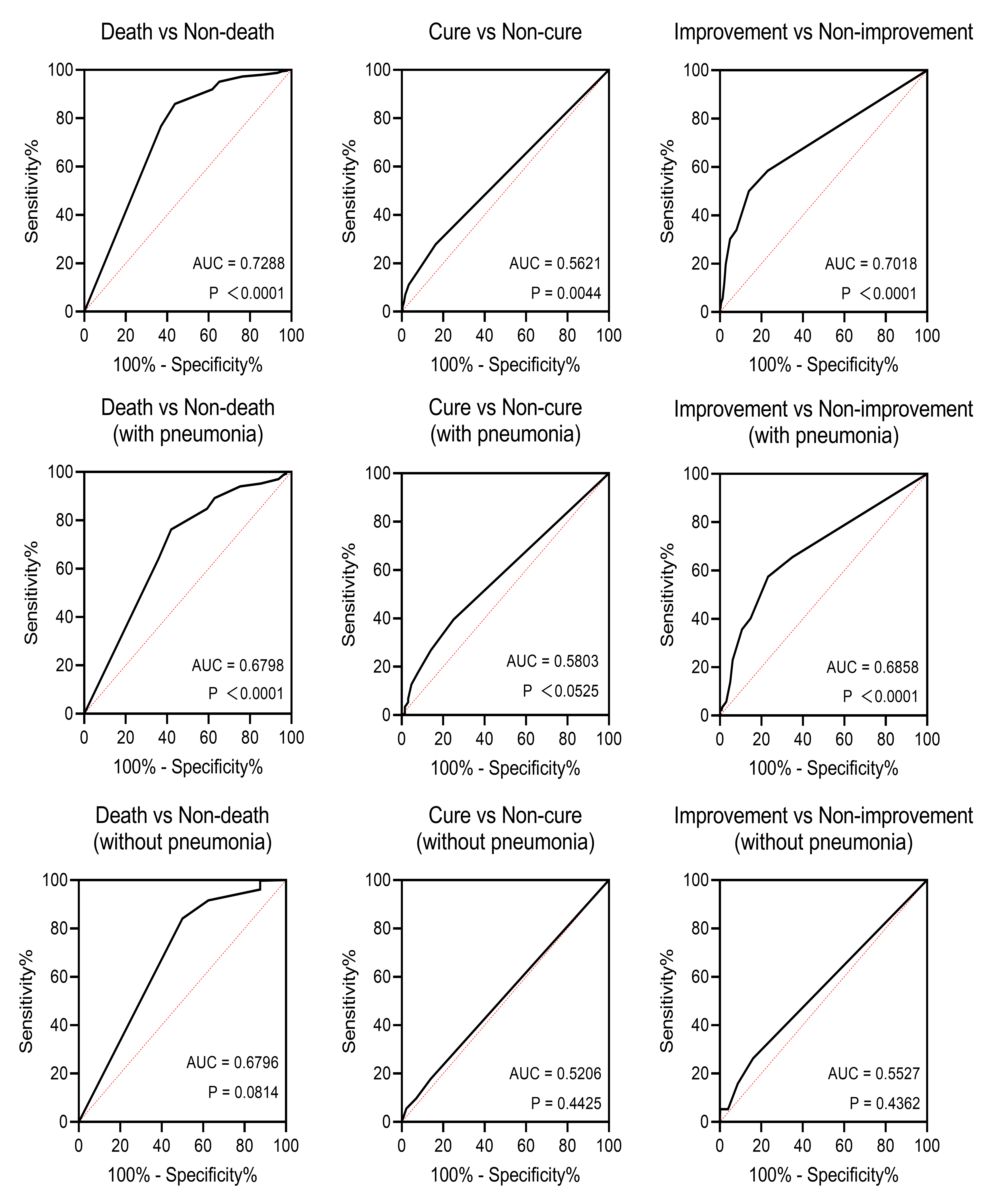


**Supplementary Figure S2. Comparison of the predictive performance of NEWS2 scores among different clinical groups of COVID-19 patients.**
